# Entropy and Complexity in QEEG Reveal Visual Processing Signatures in Autism: A Neurofeedback-Oriented and Clinical Differentiation Study

**DOI:** 10.3390/brainsci15090951

**Published:** 2025-09-01

**Authors:** Aleksandar Tenev, Silvana Markovska-Simoska, Andreas Müller, Igor Mishkovski

**Affiliations:** 1 Faculty of Computer Science and Engineering, Ss. Cyril and Methodius University of Skopje, 1000 Skopje, North Macedonia; igor.mishkovski@finki.ukim.mk; 2 Macedonian Academy of Sciences and Arts, 1000 Skopje, North Macedonia; silvana@manu.edu.mk; 3 Brain and Trauma Foundation Grison/Switzerland, Poststrasse 22, 7000 Chur, Switzerland; andreas_mueller@hin.ch

**Keywords:** Renyi entropy, Tsallis entropy, Lempel–Ziv complexity, autism, QEEG, neurofeedback

## Abstract

(1) Background: Quantitative EEG (QEEG) offers potential for identifying objective neurophysiological biomarkers in psychiatric disorders and guiding neurofeedback interventions. This study examined whether three nonlinear QEEG metrics—Lempel–Ziv Complexity, Tsallis Entropy, and Renyi Entropy—can distinguish children with autism spectrum disorder (ASD) from typically developing (TD) peers, and assessed their relevance for neurofeedback targeting. (2) Methods: EEG recordings from 19 scalp channels were analyzed in children with ASD and TD. The three nonlinear metrics were computed for each channel. Group differences were evaluated statistically, while machine learning classifiers assessed discriminative performance. Dimensionality reduction with t-distributed Stochastic Neighbor Embedding (t-SNE) was applied to visualize clustering. (3) Results: All metrics showed significant group differences across multiple channels. Machine learning classifiers achieved >90% accuracy, demonstrating robust discriminative power. t-SNE revealed distinct ASD and TD clustering, with nonlinear separability in specific channels. Visual processing–related channels were prominent contributors to both classifier predictions and t-SNE cluster boundaries. (4) Conclusions: Nonlinear QEEG metrics, particularly from visual processing regions, differentiate ASD from TD with high accuracy and may serve as objective biomarkers for neurofeedback. Combining complexity and entropy measures with machine learning and visualization techniques offers a relevant framework for ASD diagnosis and personalized intervention planning.

## 1. Introduction

Autism spectrum disorder (ASD) is a complex neurodevelopmental condition marked by persistent challenges in social communication, restricted interests, and repetitive behaviors. Despite extensive research, a complete understanding of its underlying neurobiological mechanisms remains elusive, necessitating innovative approaches to characterize the altered neural dynamics associated with the condition. The global standard for diagnosing ASD still relies on the DSM-5 and ICD-11 manuals, which take into account the behavioral characteristics of the individuals. However, the broad definition of ASD allows for diverse symptom combinations and increased heterogenity, but it reduces diagnostic precision as it blurs distinctions from other disorders, relies on subjective criteria, and risks false positives, inflated prevalence, and less targeted care [1,2]. This shift in the diagnostic criteria towards the inclusion of a larger population has addressed the need of more objective diagnosis by discovering endophenotypes that can define subtypes and explain the spectrum of the disorder [3,4]. Quantitative electroencephalography (QEEG) has emerged as a promising neuroimaging tool for investigating brain function in ASD [5], providing high temporal resolution insights into neural oscillatory patterns and connectivity networks that may be disrupted in individuals with the disorder.

Traditional QEEG analyses, primarily focusing on spectral power and coherence-based connectivity [6] may not fully capture the intricate, nonlinear dynamics inherent in neural systems. Critiques of conventional spectral analysis note that increases in band-specific power often overlap across different disorders, leading to a distorted understanding of brain function [7]. Since the human brain functions as a complex adaptive system, exhibiting emergent properties from intricate interactions between neural networks, which linear analytical methods may inadequately characterize, and given the non-stationary nature of EEG signals, nonlinear features are essential for analysis [8].

Recent advancements in complexity science and information theory have introduced sophisticated metrics capable of quantifying the irregularity, randomness, and structural complexity of EEG signals [9]. One method draws on nonlinear dynamics and chaos theory, which studies complex irregular patterns in systems governed by deterministic rules and sensitive to initial conditions. Researchers have applied complexity-based metrics to analyze brain activity, aiming to capture the intricate and unpredictable nature of neural processes. The Lyapunov coefficient, which quantifies chaotic behavior, has been used to distinguish between children with ASD and typical development (TD) [10,11,12,13]. Despite its value, this approach can be difficult to apply and often yields abstract results. A more concrete alternative involves entropy-based measures, inspired by Shannon’s information theory. Here, complexity is assessed through entropy—a concept from thermodynamics adapted to describe probabilistic systems. Entropy quantifies statistical complexity in EEG signals through uncertainty, complexity, and information content, making it suitable for analyzing the brain, a highly complex information processing system. Different entropy metrics have been developed and applied in studies that investigate EEG signals in individuals with autism, linking it to deficits in social cognition, communication, and behavior [14,15,16,17,18]. A recent study has incorporated several nonlinear complexity measures, including different notions of entropy, fractal dimensions and Lempel–Ziv Complexity, offering multi-modal view of different complexity perspectives in the analysis of EEG signals in children with autism [19].

Despite advances in complexity and nonlinear theory, its application to the analysis of autistic EEG signals has been limited to differentiation alone. There is no neurofeedback protocol developed for ASD yet, based on non-linear features, while the existing ones based on spectral characteristics, do not support evidence of the effectiveness in treatment [20]. However, it has been reported that positive effects in neurofeedback correlate with brain activation during observation [21], which involves the brain regions responsible for visual processing.

In this study, we investigate entropy-based measures such as Rényi Entropy and Tsallis Entropy to offer unique perspectives on the information content and statistical complexity of neural activity. Rényi Entropy provides a parameterizable framework for assessing information content across different scales and probability distributions. Tsallis Entropy, derived from non-extensive statistical mechanics, is particularly suited for analyzing systems with long-range correlations and non-Gaussian distributions—characteristics often observed in neural signals. Complementing these entropy measures, Lempel–Ziv Complexity (LZC) provides an algorithmic approach to quantifying the Kolmogorov complexity (randomness) of the EEG time series data. Combining these metrics, we move further from differentiation and develop explainable models that may contribute to developing neurofeedback protocol based on nonlinear metrics.

## 2. Materials and Methods

### 2.1. Data and Procedure

EEG data were collected from a total of 88 children, including 39 with typical development (TD) and 49 diagnosed with autism spectrum disorder (ASD). The average age of the ASD group was 6.18 years (SD = 1.98), while the TD group had a mean age of 5.35 years (SD = 2.31). Since all participants in the TD group were male, gender was excluded from further analysis due to the absence of a balanced gender distribution between the groups. ASD diagnoses were confirmed jointly by a psychiatrist and a psychologist, with inclusion contingent upon agreement between both professionals. All children in the ASD group met the diagnostic criteria outlined in the DSM-V. To enhance the diagnostic reliability, additional background information was obtained from parents. All EEG recordings were conducted in Skopje, North Macedonia. Informed written consent was obtained from the parents of all participants. The study received ethical approval from the Ethics Committee of the Faculty of Medicine at Ss. Cyril and Methodius University in Skopje (Reference No. 03-4953/2).

Due to the characteristics of the disorder, EEG recordings were conducted in a resting-state condition with participants’ eyes open. Electrode placement followed the international 10/20 system using an Electro-Cap provided by Electro-Cap International. EEG activity was recorded from 19 channels: Fp1, Fp2, F3, F4, F7, F8, Fz, C3, C4, Cz, T3, T4, T5, T6, P3, P4, Pz, O1, and O2, with linked ears serving as the reference. The ground electrode was positioned between Fpz and Fz.

To monitor and control for eye movement artifacts, electrooculogram (EOG) signals were recorded using two 9 mm tin electrodes placed above and below the right eye, referenced to Fpz and Oz. An artifact rejection threshold of 50 μV was applied. The amplifier settings included a low-frequency filter at 0.53 Hz, a high-frequency filter at 50 Hz, and a notch filter between 45–55 Hz. Electrode impedance was maintained below 5 kΩ. EEG signals were digitized at a sampling rate of 250 Hz and stored for offline analysis using Mitsar WinEEG software, version 2.103.70.

For the TD group, continuous EEG segments of 3 min were obtained, while the ASD group recordings ranged from 7 to 30 min, depending on the child’s tolerance and behavior during the session. The variability in recording length for the ASD group was due to the challenges of maintaining a calm environment. The second author monitored the sessions in real time, taking notes and visually inspecting the recordings to determine the presence of artifacts and the acceptability of each segment in later offline preprocessing analysis.

All EEG recordings were identically preprocessed. Initially, ocular artifacts were corrected using the Mitsar WinEEG software by nullifying the activation curves of individual independent component analysis (ICA) components associated with eye blinks. Furthermore, EEG epochs were automatically excluded from further analysis if they exhibited excessive amplitude (greater than 100 μV), or if they contained abnormally fast (greater than 35 μV in the 20–35 Hz band) or slow (greater than 50 μV in the 0–1 Hz band) frequency activity. Finally, each EEG was manually inspected to verify artifact removal. For time-domain analysis, the longest continuous artifact-free segment during which the child was quietly seated was selected. To ensure consistency across participants, all segments were trimmed to match the shortest maximum usable length across subjects—43 s. We did not segment the 43 s time-frame into smaller windows while computing the metrics, but instead used the whole segment in our analysis, since the chosen metrics described below offer different but complementary perspectives that could still provide meaningful insights. Given the 250 Hz sampling rate, this resulted in 10,750 data points per channel, totaling 204,250 amplitude values per subject across the 19 channels.

### 2.2. Metrics

The Lempel–Ziv Complexity (LZC), Renyi Entropy (RE), and Tsallis Entropy (TE) were chosen for analyzing electroencephalogram (EEG) signals in children with autism spectrum disorder (ASD) due to their ability to provide unique insights into brain dynamics. LZC is a valuable metric because it measures the randomness in a signal by calculating the number of distinct substrings in a binary sequence derived from the EEG signal [22]. A key advantage of LZC is its robustness to noise and artifacts, and it does not require prior assumptions about the EEG signal’s underlying structure or dynamics. Renyi Entropy, as a generalization of Shannon entropy, offers flexibility in analysis through an adjustable parameter that highlights different aspects of the amplitude distribution within the signal [23]. It effectively captures amplitude variations and distributions across the signal, with higher values in ASD suggesting a “noisier” signal and increased complexity or disorder in brain dynamics. Tsallis Entropy further generalizes entropy, proving useful for analyzing non-extensive systems. It can capture complexity and nonlinearity in systems that are not well-described by traditional Boltzmann–Gibbs entropy, including those with long-range interactions, memory, or fractal structures [24]. Additionally, Tsallis Entropy is adept at capturing non-stationarity and non-Gaussian characteristics of EEG signals and can be computed efficiently and robustly, which is beneficial for quantifying changes in EEG signals corresponding to different brain states and for machine learning classification. Together, these metrics offer a comprehensive view of the inherent irregularity, statistical order, and predictability of EEG signals, contributing to the identification of neurophysiological differences associated with ASD.

#### 2.2.1. Renyi Entropy

**Figure 1:**
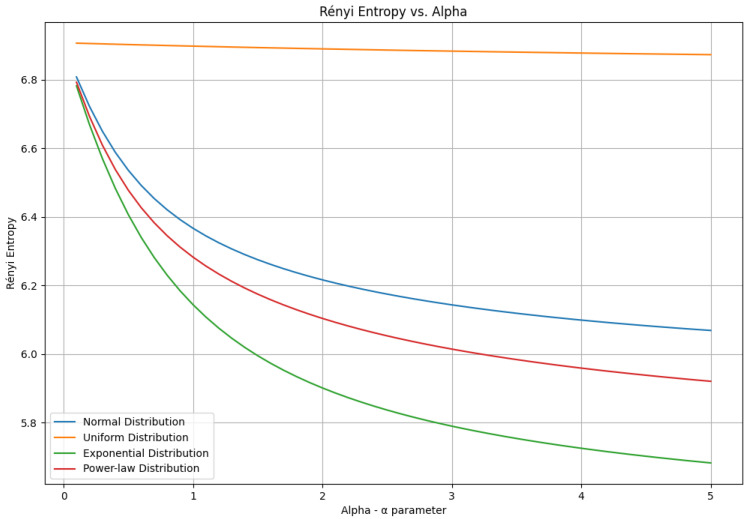
Simulation of the value of Rényi Entropy depending on the value of the parameter α. We observe that the value of the entropy represents a logarithmically convex function, i.e., it converges and does not increase depending on the parameter α. Additionally, from the figure, we see that the value of the entropy depends more on the probability distribution function of the sample than on the choice of α.

Rényi Entropy of the EEG signal can be computed using segmentation methods, such as wavelet packet transformation or symbolic dynamics [25,26]. We used kernel density estimation to estimate the probability function of the EEG signal [27].

(1) $$H_{\alpha }\left(f\right)=\frac{1}{1-\alpha }\log\int_{-\infty }^{\infty }f{\left(x\right)}^{\alpha }dx,$$

where f(x) is the probability density function, and α>0, α≠1. This is a novel method for computing Rényi Entropy of a continuous variable, as it avoids the issue of binarizing the data into discrete intervals. Rényi Entropy is not a simple function of the EEG signal length; rather, it depends on how the EEG signal is segmented or symbolized. Different segmentation methods can lead to different Rényi Entropy values, even for the same EEG signal and the same α. Therefore, Rényi Entropy is not a universal measure of the information content of the EEG signal, but a relative measure that depends on the choice of segmentation scheme (Figure 1).

It depends on the parameter α, which can be adjusted to yield different entropy measures. For example, when α=1, Rényi Entropy reduces to Shannon entropy, and when α=∞, Rényi Entropy becomes the minimum entropy (min-entropy), which represents the negative natural logarithm of the probability of the most likely outcome.

According to the definition and as seen from Figure 1, if 0<α<1, emphasis is placed on rare events in the signal (higher values of entropy). Since, in our analysis, we do not want to emphasize rare occurrences in the EEG signals, we chose a value of α>1 or α=2, which emphasizes frequent occurrences in the EEG signals or patterns characteristic of typically developing children or children with autism. It is reasonable to consider that the behavioral differences observed in children, as seen in their clinical behavioral characteristics, are not the result of random disturbances in brain dynamics and activity but rather the result of systemic differences.

#### 2.2.2. Tsallis Entropy

Tsallis Entropy is a generalized entropy measure that is defined as follows:

(2) $$S_{q}=\frac{1}{q-1}\left(1-\sum_{i=1}^{n}p_{i}^{q}\right),$$

where *n* is the number of possible outcomes, $p_{i}$ is the probability of the *i*-th outcome, and q≠1 is the entropic index.

Tsallis Entropy introduces a parameter *q*, which controls the degree of nonextensivity or nonadditivity in the system. When q=1, Tsallis Entropy simplifies to the classical Boltzmann–Gibbs entropy. For values of q>1, the entropy becomes sub-extensive, indicating that the total entropy of a system is less than the sum of its parts. Conversely, when q<1, the entropy is super-extensive, meaning the total entropy exceeds the sum of the individual components. This entropy measure is particularly useful for analyzing EEG signals, as it can capture dynamic changes associated with different brain states. It also serves as a valuable feature in machine learning and classification tasks. Compared to traditional entropy measures, Tsallis Entropy offers several advantages: it effectively models non-Gaussian distributions, handles nonstationary signals, and reflects the multi-scale characteristics of EEG data. Additionally, it can be computed efficiently and is robust to noise and variability.

We conducted a sensitivity analysis (Table 1 and Table 2) of the parameter q∈{x∈[−2,2]∣x=−2+0.1k, k∈Z, x≠1} and chose the value of q=1.5 when building our machine learning models. The explanation is the same as with Renyi Entropy, to emphasize frequent events with q>1 in the signal [28,29]. From Table 1 and Table 2, we can also note the significant difference between the TD and ASD groups for every *q* value, confirming the robustness of the metric.

#### 2.2.3. Lempel–Ziv Complexity

Lempel–Ziv Complexity (LZC) is a metric used to quantify the repetitiveness or structural complexity of binary sequences and text. It is grounded in a basic principle of identifying and copying unique patterns or “words” within a sequence. This measure is considered flexible and effective, as it meets key expectations for a complexity metric: sequences with regular patterns yielding low complexity values, while longer and more irregular sequences result in higher complexity. LZC is calculated by counting the number of distinct substrings encountered as the binary sequence is scanned from left to right. For instance, the binary string 010101010101 has low complexity because it contains only two repeating patterns: 01 and 10. In contrast, the string 011001011110 has higher complexity, with six unique substrings: 0, 1, 01, 10, 100, and 111.

One of the main advantages of LZC is that it does not require prior assumptions or models about the underlying structure or dynamics of the EEG signal. Additionally, it is robust to noise and artifacts, making it a practical tool for analyzing complex biological signals. In our study, we first converted the EEG signal into a binary sequence using a thresholding method. The threshold was set to the mean amplitude of the signal. Segments with amplitudes above the threshold were assigned a value of 1, while those below were assigned 0.

### 2.3. Machine Learning Methods

To translate these complexity-based neurophysiological findings into clinically applicable diagnostic tools, machine learning approaches offer powerful methods for pattern recognition and classification. In this study, we employed multiple complementary machine learning algorithms to develop robust classification models based on the extracted complexity features. Support vector machines (SVM) utilize hyperplane optimization to find optimal decision boundaries in high-dimensional feature spaces, making them particularly suitable for biomedical classification tasks with complex nonlinear relationships. Artificial neural networks (ANN) employ interconnected processing units that mimic biological neural networks, enabling the learning of intricate patterns and nonlinear mappings between input features and diagnostic outcomes. The J48 algorithm implements the C4.5 decision tree approach, constructing interpretable tree-based models that provide transparent decision rules through recursive feature splitting based on information gain criteria. REPTree (Reduced Error Pruning Tree) generates fast decision trees using reduced-error pruning techniques, offering computational efficiency while maintaining classification accuracy through systematic overfitting reduction. Random gorest combines multiple decision trees through ensemble learning, utilizing bootstrap aggregation and random feature selection to improve generalization performance and reduce overfitting while providing feature importance rankings. By implementing this diverse array of machine learning approaches, we aim to identify the most effective algorithmic framework for autism classification based on QEEG complexity measures, while ensuring robust model validation and clinical interpretability.

The present study aims to investigate the neural complexity profiles in individuals with autism using a comprehensive QEEG analysis framework incorporating Rényi Entropy, Tsallis Entropy, and Lempel–Ziv Complexity measures. Through systematic comparison of these metrics between autism and neurotypical control groups, coupled with advanced machine learning classification approaches, we seek to identify specific patterns of altered neural complexity that may serve as neurobiological signatures of autism spectrum disorder.

## 3. Results

**Figure 2:**
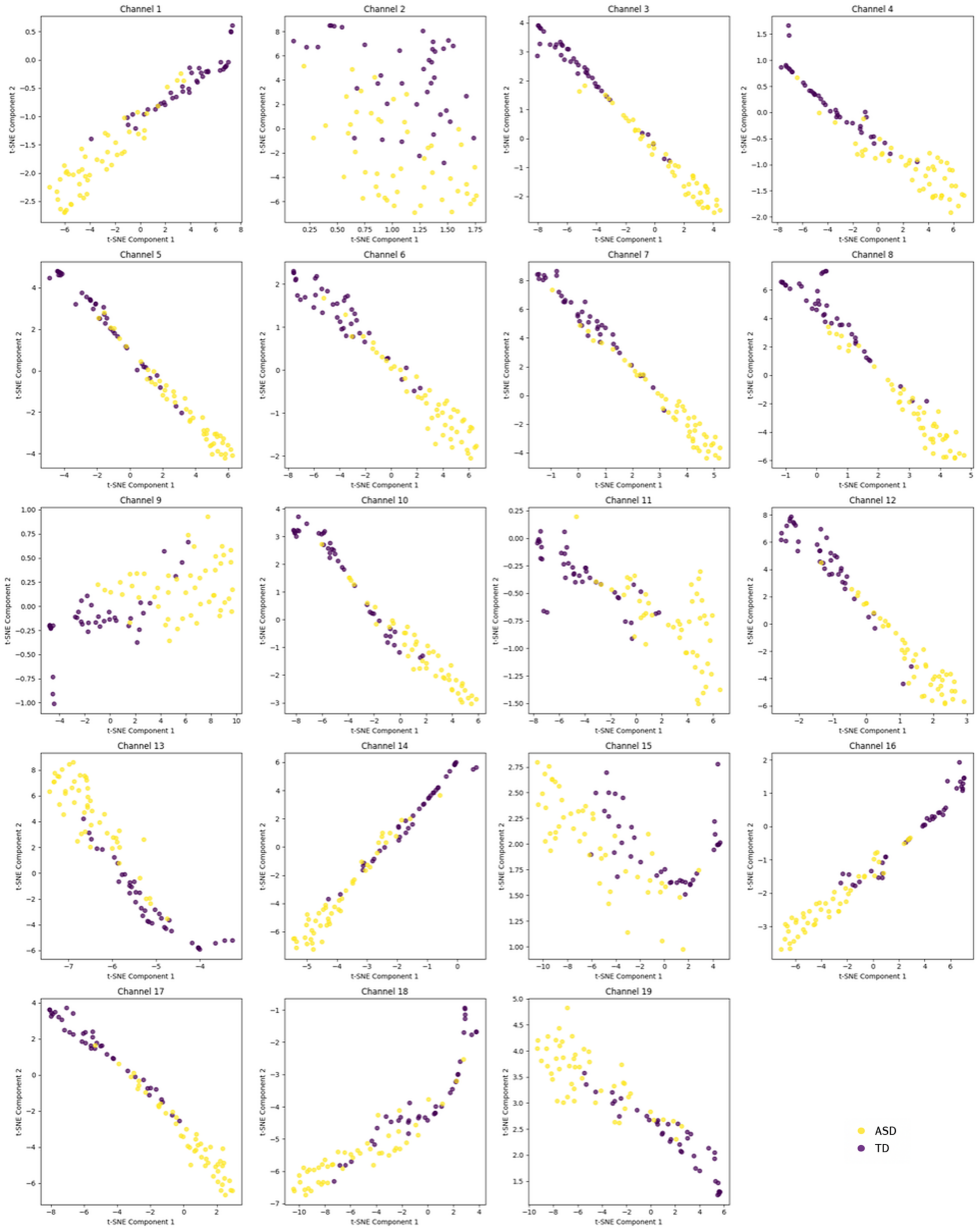
Clustering results of the samples in space based on the features: Rényi Entropy, Tsallis Entropy, and Lempel–Ziv Complexity, using the t-SNE technique for each channel separately.

**Figure 3:**
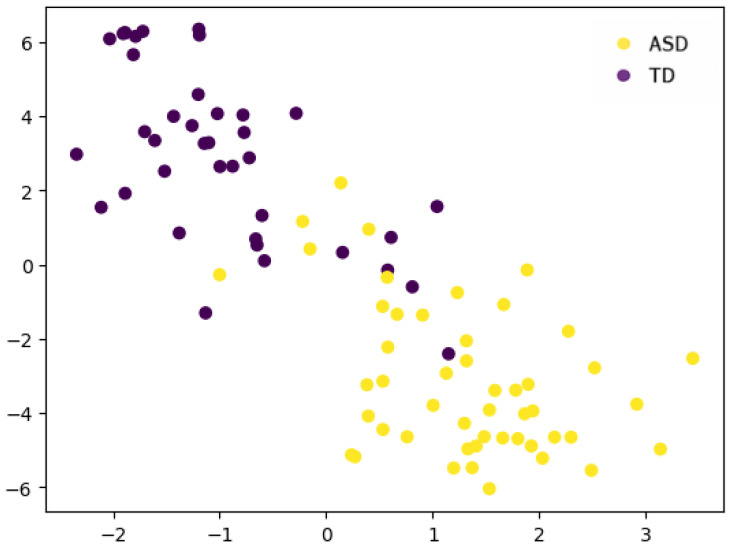
Clustering results of the samples in space based on the features: Rényi Entropy, Tsallis Entropy, and Lempel–Ziv Complexity, using the t-SNE technique after concatenating all individual channels.

The models were trained using a total of 3 ∗ 19 = 57 attributes, corresponding to the values of the three metrics across the 19 EEG channels. Before training the models, we used all the features computed from a single channel and visualized the subjects in 2D using the t-SNE technique. t-SNE is a nonlinear dimensionality reduction technique that converts pairwise distances in high-dimensional space into conditional probabilities representing similarities. It then embeds the data in a low-dimensional space by minimizing the Kullback–Leibler divergence between the high-dimensional similarity distribution and a Student-t distribution over pairwise distances in the embedding via gradient descent. This approach excels at preserving local neighborhood structure and revealing cluster patterns, though absolute global distances may not be accurately represented. The results of the t-SNE technique are presented in Figure 2 for each channel separately. Additionally, by concatenating the features from the individual channels and reapplying t-SNE, the result shown in Figure 3 was obtained, indicating an almost perfect clustering.

Table 3 shows the labeling for the EEG channels used in the analysis.

Using this technique, it is shown that regardless of the channel, the features are powerful enough to distinguish between the control and ASD subjects. An interesting finding is that the features from the 2nd, 9th, 11th, and 15th channels, corresponding to the electrodes Fp2, C3, C5, and Pz, introduce some prominent nonlinear patterns, further suggesting that the features from these channels could be used with nonlinear machine learning techniques, depending on the learning scenario being addressed.

**Figure 4:**
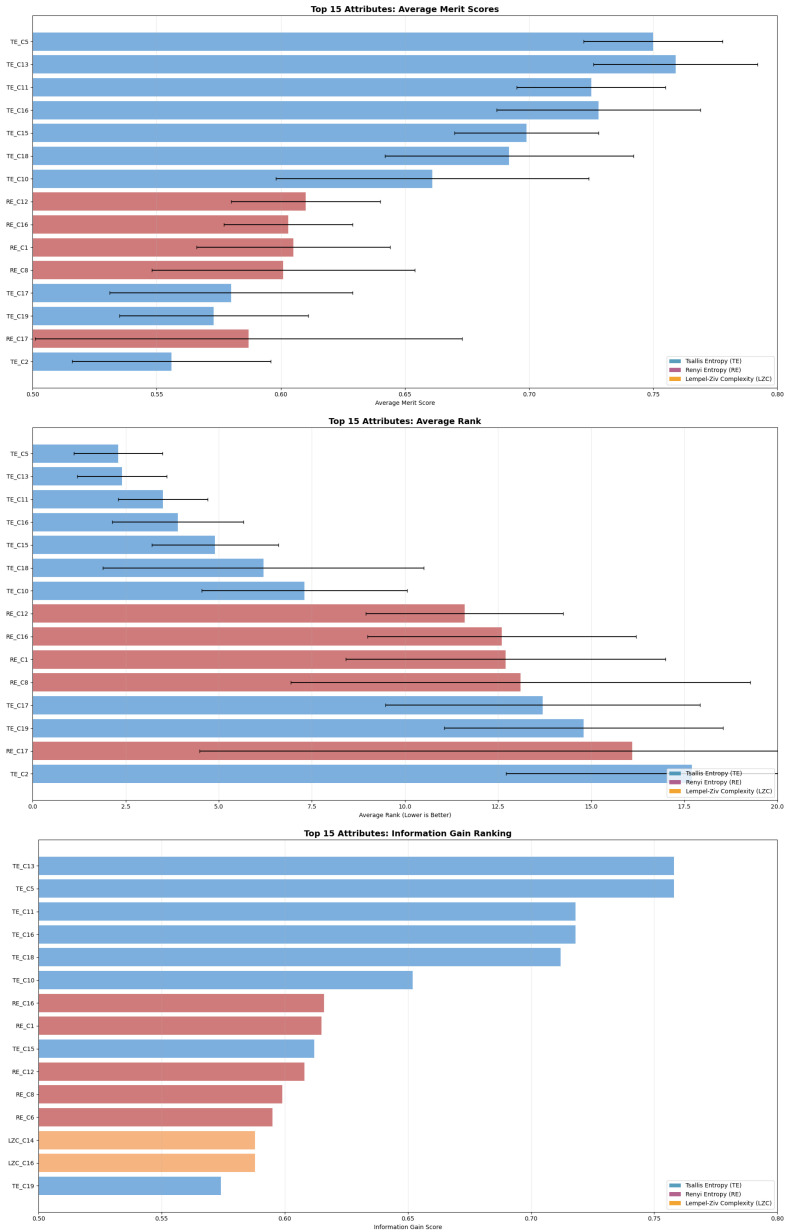
Attribute evaluation based on information gain metric. Only the top 15 attributes are shown. The top five best attributes correspond to the value of Tsallis Entropy in channels 5, 13, 11, 16, and 15 corresponding to electrodes Fz, T5, C4, P4, and Pz accordingly, suggesting importance in signatures in the frontal, temporal, central, and parietal regions.

**Figure 5:**
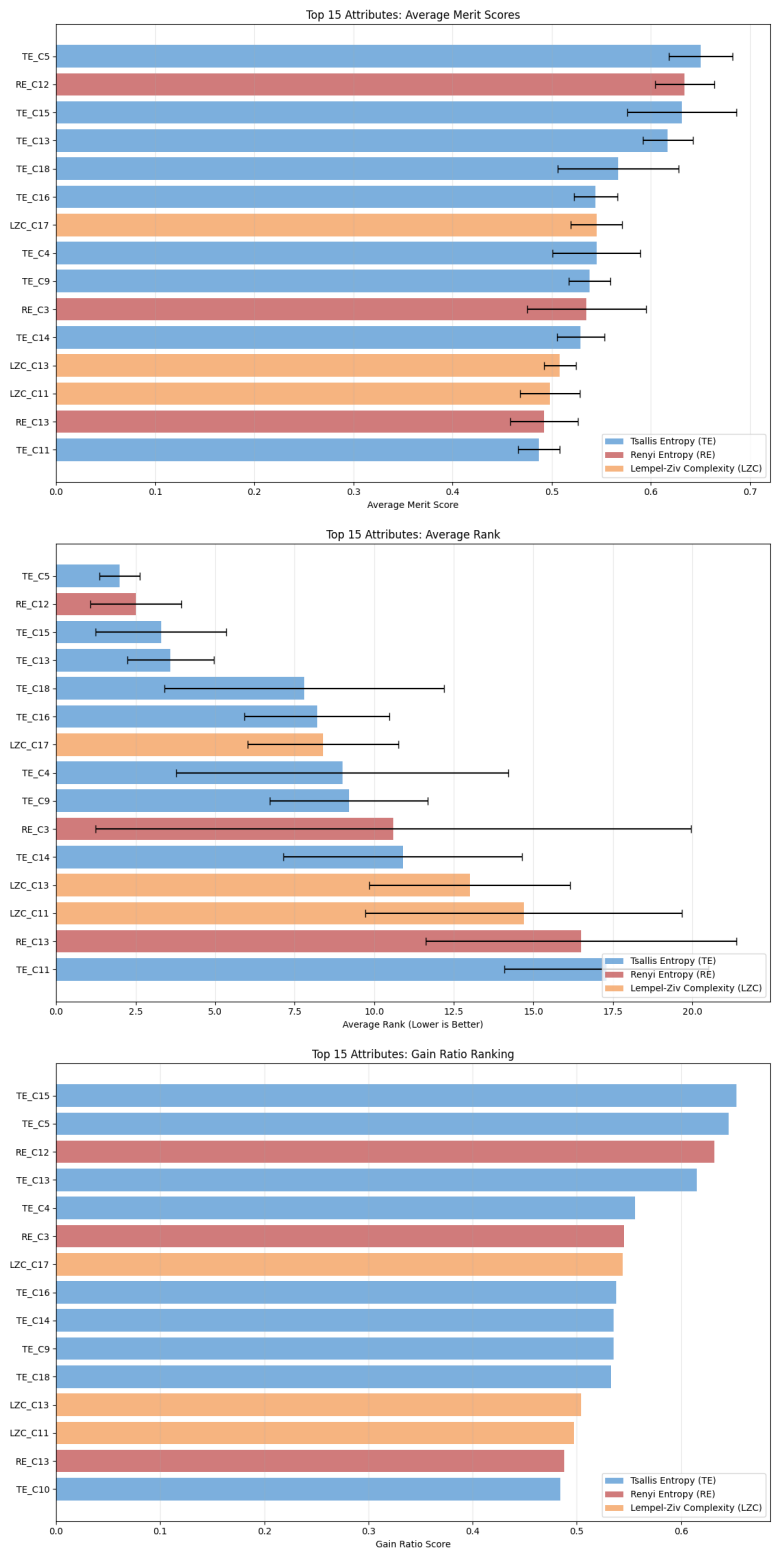
Attribute evaluation based on gain ratio metric. Only the top 15 attributes are shown. The top five best attributes correspond to the value of Tsallis Entropy in channels 5, 15, 13, and 18 corresponding to electrodes Fz, Pz, T5, and O1 accordingly and the value of Renyi Entropy in channel 12, coresponding to electrode T4, suggesting importance in signatures in the frontal, temporal, parietal, and ocipital regions.

Before training the models, we used two techniques for attribute evaluation, the InfoGainAtributeEvaluation algorithm (Figure 4) and the GainRatioAttributeEvaluation algorithm (Figure 5) to detect which attributes suggest importance. This analysis is complementary to the statistical analysis that showed statistical significance in all electrode channels between the two groups of children. With this, we can extract additional knowledge about the specific regions that contribute the most. Based on the attribute importance, we tested the models in four different setups, based on the gain-ratio (Figure 5), to check their performance: (1) training with all 57 attributes; (2) training with the top 15 attributes; (3) training with the top 10 attributes; and (4) training with the top 5 attributes. Random forest performed with the same accuracy in all the setups, while the neural network and SVM decreased in performance as we excluded and lowered the number of attributes. J48 had little change in performance, achieving 96.6% accuracy in the setup with 57 attributes and the setup with the top 5 attributes, while showing a lower accuracy of 95.5% in the other two setups. The RepTree algorithm had 92% accuracy in the setup with all attributes and 96.6% in the other three setups.

The evaluation results of the models are shown in Table 4, Table 5, Table 6, Table 7 and Table 8 for the algorithms: random forest, decision tree (J48), support vector machine (SVM), decision tree (RepTree), and artificial neural network, respectively.

**Figure 6:**
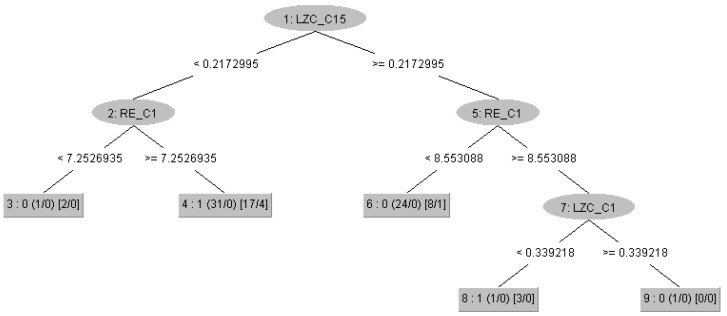
Decision tree obtained using the RepTree algorithm with all of the 57 attributes. The root of the tree is the value of the Lempel–Ziv Complexity (LZC) in the Pz channel. The tree consists of a total of nine nodes (five leaves and four branching nodes). At the first level of the tree are nodes comparing the values of Rényi Entropy (RE) in the Fp1 channel. At the second level, there is another node that branches the tree depending on the LZC value in the Fp1 channel. The number before the small brackets indicates the class (0—Control, 1—ASD). The small brackets at the leaves show the ratio of the total number of children in that leaf to the misclassified children during the model training phase. The medium brackets show the ratio of the total number of children in that leaf to the misclassified children during the tree pruning phase.

**Figure 7:**
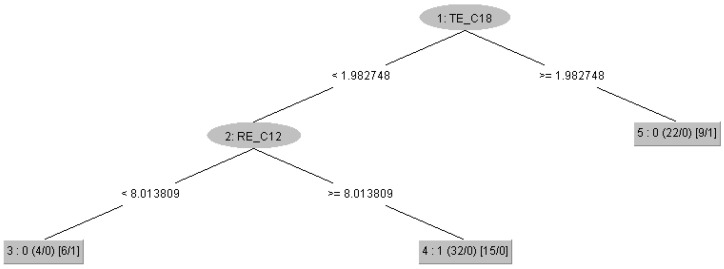
Decision tree obtained using the RepTree algorithm with only the top five attributes based on the gain ratio. The root of the tree is the value of Tsallis Entropy (TE) in the O1 channel. The tree consists of a total of five nodes (three leaves and two branching nodes). At the first level of the tree is a node comparing the value of the Rényi Entropy (RE) in the T4 channel. The number before the small brackets indicates the class (0—Control, 1—ASD). The small brackets at the leaves show the ratio of the total number of children in that leaf to the misclassified children during the model training phase. The medium brackets show the ratio of the total number of children in that leaf to the misclassified children during the tree pruning phase.

**Figure 8:**
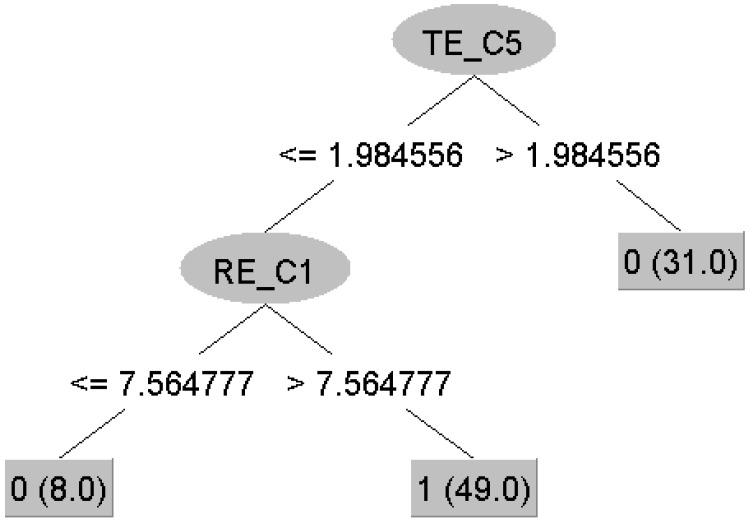
Decision tree obtained using the J48 algorithm. The root of the tree is the value of the Tsallis Entropy (TE) in the Fz channel. The tree consists of a total of five nodes (three leaves and two branching nodes). At the first level of the tree is a node that checks the value of the Rényi Entropy (RE) in the Fp1 channel. The number before the small brackets indicates the class (0—Control, 1—ASD). The brackets at the leaves show the total number of children belonging to that leaf during the model training phase.

All models perform excellently, with the random forest algorithm standing out as the best with an accuracy of 97.7%, followed by the J48, RepTree, and SVM (96.6%) and the neural network (94.3%). This result confirms that metrics such as the Rényi and Tsallis Entropies and Lempel–Ziv Complexity can be used as biomarkers or endophenotypes in autism diagnosis. Additionally, one of the advantages of decision trees is that the classification model is easy to interpret. In our case, the decision trees appear as shown in Figure 6, Figure 7 and Figure 8.

## 4. Discussion

Biologically speaking, the processing of visual signals in the brain (important for the resting state with eyes open) occurs in several stages, and different brain regions cooperate in this process. The light signal, through the retina in the eye, activates the optic nerve, which generates an electrical signal that travels to the thalamus. The thalamus forwards the signal to the occipital region of the brain (electrodes 18 and 19, i.e., O1 and O2). From there, the signal follows two pathways: (1) the dorsal pathway (“where”) to the parietal part of the brain (electrodes 14, 15, and 16, i.e., P3, Pz, and P4), responsible for recognizing movement in space and overall spatial orientation, and (2) the ventral pathway (“what”) to the temporal part of the brain (electrodes 8, 12, 13, and 17, i.e., T3, T4, T5, and T6), responsible for recognizing shapes, faces, and colors. Finally, the signal reaches the frontal part (electrodes 1, 2, 4, 5, and 6, i.e., Fp1, Fp2, F3, Fz, and F4), where the brain’s main processing functions occur, including visual working memory, attention, and decision-making. The integration of results from all brain regions takes place in the central part (electrodes 9, 10, and 11, i.e., C3, Cz, and C4), which is crucial during the resting phase, meaning the brain is awake and ready to respond at any moment. It has been suggested that behavioral sex differences in visual motion perception are generated, and one should consider sex as a crucial biological variable in both human brain and behavioral research [30]. Therefore, we acknowledge that all participants in the TD group were male, resulting in an imbalance across the study groups. This limitation may influence the generalization of our findings, particularly in light of the established sex differences in ASD presentation and neurobiological profiles. Although our decision to exclude gender from further analysis was based on the lack of variability in the TD group, the identified biomarkers still reflect patterns even if they may not fully capture the broader phenotype of ASD, especially in females. Future studies should aim to include balanced data to better confirm the results. We also acknowledge that the ASD group was slightly older than the TD group (6.18 vs. 5.35 years), which may introduce a potential confounding effect. Although the age difference is relatively small, developmental changes in EEG characteristics during early childhood could influence the results.

With the results of Figure 2, we highlighted the nonlinearity in the clustering of subjects at electrodes 2, 9, 11, and 15, which are involved in the various processes of the brain dynamics described above during rest. This nonlinearity confirms, as expected, that something is happening in those brain regions. In a different study of power spectra that used the same dataset, the authors found limited or no activation in the ASD group in C3 and C4 in the alpha band, crucial for the resting state and indicating awareness [19]. With these results, we prove that this is not true and there exists an activation in these regions; however, there may be impairment of the structural connectivity in an autistic brain like less radial oriented neurons or maybe over-connectivity in the region, so that the information diffuses across the neural network and is not captured by spectral analysis, proving again that time-domain analysis is important. On the other hand, the machine learning models built using decision trees and shown in Figure 6 and Figure 8, again point to regions involved in the entire process, which are key in training the models (electrodes 1, 5, and 15). The differences observed between subjects from the two groups indicate impaired functional connectivity between the affected brain regions in children with ASD.

## 5. Conclusions

From the machine learning results, we can draw three main conclusions. First, Figure 2 and Figure 3 confirm, as mentioned in the Results section, that the choice of metrics is so effective in spatially clustering the subjects that the machine learning task becomes trivial, and we can even say that it may not be necessary at all—simple statistical analysis is sufficient to distinguish between TD and ASD, and the metrics can be used as potential biomarkers for ASD. Therefore, the good performance of the machine learning models was expected. Second, from a machine learning perspective, the nonlinear patterns present in the signals from the electrodes are best learned through neural networks. Although decision trees contain nonlinearity and are nonlinear methods themselves, the best mathematical operator or function for learning nonlinear patterns is the artificial neural network. Thus, we can confidently say that for a sufficiently large dataset, neural networks are the best choice for this problem, even though in our case ANN showed the “worst” performance. Finally, as a third conclusion, we believe that in clinical practice, a neurofeeback protocol can be optimized using the electrodes of interest. Because decision trees provide clear rule-based boundaries, they allow for personalized and interpretable neurofeedback goals, which are crucial for clinical applicability and user engagement. The decision tree structure (for example, LZC_C15≤0.2173) offers interpretable thresholds that can be utilized. In this case, LZC reflects the randomness of EEG signals at channel Pz (C15), located in the parietal zone, which is associated with sensory integration and attentional processes. A lower LZC value suggests reduced signal complexity, a more regular signal, or a less random signal. In a neurofeedback context, this threshold can serve as a target for training: individuals could be guided to increase the complexity of their parietal EEG activity (i.e., raise LZC_C15 above 0.2173) through real-time feedback. This could be achieved using visual or auditory cues that reward increased complexity, promoting more random neural patterns. The same approach holds with the results from other decision tree nodes (Fp1 and Fz electrodes), with the only difference between the metrics, since in the context of entropy, the protocol should stimulate decreasing the entropy values and reducing the neural noise.

## Figures and Tables

**Table 1:** Sensitivity analysis of entropic index *q* with Mann–Whitney U test: part 1. The table values represent the *p*-values of the statistical test of significance between the groups of TD and ASD subjects in each EEG channel. The significance level is 0.05, but we addressed the multiple comparison problem using Bonferoni correction. The corrected significance level was computed by simply dividing 0.05 with 19, corresponding to 19 electrode channels, or 0.0026.

Entropic Index	Fp1	Fp2	F7	F3	Fz	F4	F8	T3	C3
q=−2	0.0094	0.0117	0.0007	0.0114	0.0114	0.0906	0.0319	0.0124	0.0207
q=−1.9	0.0094	0.0105	0.0007	0.0085	0.0100	0.0790	0.0297	0.0124	0.0173
q=−1.8	0.0105	0.0097	0.0007	0.0074	0.0103	0.0714	0.0264	0.0134	0.0169
q=−1.7	0.0092	0.0097	0.0008	0.0078	0.0092	0.0631	0.0228	0.0153	0.0130
q=−1.6	0.0087	0.0108	0.0010	0.0058	0.0089	0.0429	0.0187	0.0178	0.0105
q=−1.5	0.0076	0.0097	0.0010	0.0049	0.0094	0.0392	0.0149	0.0178	0.0100
q=−1.4	0.0074	0.0097	0.0011	0.0051	0.0097	0.0270	0.0114	0.0169	0.0085
q=−1.3	0.0071	0.0094	0.0011	0.0037	0.0071	0.0192	0.0100	0.0130	0.0068
q=−1.2	0.0076	0.0089	0.0012	0.0033	0.0064	0.0169	0.0094	0.0100	0.0049
q=−1.1	0.0058	0.0076	0.0016	0.0033	0.0055	0.0130	0.0069	0.0080	0.0037
q=−1	0.0048	0.0055	0.0021	0.0030	0.0035	0.0094	0.0069	0.0078	0.0030
q=−0.9	0.0028	0.0046	0.0021	0.0024	0.0030	0.0064	0.0052	0.0068	0.0027
q=−0.8	0.0025	0.0040	0.0024	0.0018	0.0027	0.0048	0.0039	0.0062	0.0021
q=−0.7	0.0021	0.0040	0.0024	0.0014	0.0021	0.0029	0.0030	0.0054	0.0021
q=−0.6	0.0016	0.0033	0.0028	0.0012	0.0019	0.0024	0.0023	0.0048	0.0019
q=−0.5	0.0016	0.0030	0.0024	0.0011	0.0016	0.0021	0.0019	0.0040	0.0019
q=−0.4	0.0015	0.0026	0.0019	0.0010	0.0014	0.0020	0.0018	0.0037	0.0015
q=−0.3	0.0013	0.0018	0.0018	0.0010	0.0013	0.0015	0.0015	0.0037	0.0014
q=−0.2	0.0010	0.0016	0.0015	0.0010	0.0012	0.0014	0.0014	0.0032	0.0012
q=−0.1	0.0010	0.0011	0.0011	0.0009	0.0009	0.0013	0.0012	0.0015	0.0009
q=0	0.0009	0.0009	0.0009	0.0009	0.0009	0.0009	0.0009	0.0009	0.0009
q=0.1	0.0009	0.0007	0.0008	0.0008	0.0008	0.0008	0.0008	0.0007	0.0008
q=0.2	0.0011	0.0005	0.0008	0.0007	0.0008	0.0006	0.0005	0.0005	0.0007
q=0.3	0.0014	0.0004	0.0007	0.0006	0.0007	0.0006	0.0005	0.0005	0.0005
q=0.4	0.0034	0.0003	0.0008	0.0005	0.0006	0.0005	0.0006	0.0003	0.0005
q=0.5	0.0040	0.0003	0.0014	0.0005	0.0005	0.0005	0.0007	0.0003	0.0003
q=0.6	0.0045	0.0002	0.0024	0.0005	0.0004	0.0005	0.0007	0.0002	0.0003
q=0.7	0.0035	0.0002	0.0035	0.0005	0.0003	0.0005	0.0006	0.0002	0.0002
q=0.8	0.0029	0.0001	0.0037	0.0005	0.0003	0.0004	0.0008	0.0001	0.0001
q=0.9	0.0040	0.0001	0.0074	0.0006	0.0002	0.0004	0.0007	0.0001	0.0001
q=1.1	0.0108	0.0001	0.0157	0.0010	0.0001	0.0005	0.0010	0.0000	0.0000
q=1.2	0.0197	0.0001	0.0149	0.0010	0.0001	0.0007	0.0015	0.0000	0.0000
q=1.3	0.0350	0.0001	0.0124	0.0010	0.0001	0.0010	0.0029	0.0000	0.0000
q=1.4	0.0458	0.0001	0.0117	0.0008	0.0001	0.0009	0.0029	0.0000	0.0000
q=1.5	0.0593	0.0001	0.0097	0.0009	0.0001	0.0010	0.0023	0.0000	0.0000
q=1.6	0.0872	0.0001	0.0094	0.0007	0.0000	0.0010	0.0021	0.0000	0.0000
q=1.7	0.1096	0.0001	0.0130	0.0009	0.0000	0.0010	0.0018	0.0000	0.0000
q=1.8	0.1387	0.0001	0.0169	0.0008	0.0000	0.0010	0.0018	0.0000	0.0000
q=1.9	0.1594	0.0000	0.0197	0.0007	0.0000	0.0012	0.0023	0.0000	0.0000
q=2	0.1764	0.0001	0.0202	0.0007	0.0000	0.0014	0.0024	0.0000	0.0000

**Table 2:** Sensitivity analysis of entropic index *q* with Mann-Whitney U test: part 2. The table values represent the *p*-values of the statistical test of significance between the groups of TD and ASD subjects in each EEG channel. The significance level is 0.05, but we addressed the multiple comparison problem using Bonferoni correction. The corrected significance level was computed by simply dividing 0.05 with 19, corresponding to 19 electrode channels, or 0.0026.

Entropic Index	Cz	C4	T4	T5	P3	Pz	P4	T6	O1	O2
q=−2	0.0700	0.0283	0.0024	0.0141	0.0182	0.0350	0.0051	0.0169	0.0468	0.0319
q=−1.9	0.0618	0.0240	0.0026	0.0124	0.0173	0.0342	0.0046	0.0192	0.0392	0.0311
q=−1.8	0.0556	0.0207	0.0025	0.0082	0.0134	0.0311	0.0041	0.0192	0.0358	0.0223
q=−1.7	0.0458	0.0192	0.0024	0.0066	0.0117	0.0234	0.0036	0.0207	0.0264	0.0157
q=−1.6	0.0334	0.0173	0.0023	0.0060	0.0087	0.0153	0.0029	0.0212	0.0207	0.0127
q=−1.5	0.0223	0.0178	0.0021	0.0062	0.0076	0.0124	0.0022	0.0192	0.0165	0.0103
q=−1.4	0.0182	0.0169	0.0019	0.0058	0.0068	0.0097	0.0019	0.0197	0.0124	0.0087
q=−1.3	0.0134	0.0137	0.0022	0.0052	0.0064	0.0080	0.0020	0.0182	0.0103	0.0066
q=−1.2	0.0127	0.0094	0.0022	0.0064	0.0055	0.0074	0.0021	0.0169	0.0074	0.0055
q=−1.1	0.0114	0.0071	0.0019	0.0048	0.0040	0.0068	0.0019	0.0108	0.0055	0.0040
q=−1	0.0092	0.0054	0.0018	0.0030	0.0035	0.0060	0.0019	0.0085	0.0040	0.0031
q=−0.9	0.0069	0.0035	0.0019	0.0025	0.0025	0.0054	0.0018	0.0068	0.0029	0.0025
q=−0.8	0.0052	0.0027	0.0016	0.0020	0.0021	0.0037	0.0015	0.0046	0.0025	0.0022
q=−0.7	0.0046	0.0019	0.0017	0.0017	0.0020	0.0036	0.0015	0.0030	0.0022	0.0021
q=−0.6	0.0035	0.0017	0.0015	0.0016	0.0017	0.0030	0.0014	0.0027	0.0020	0.0019
q=−0.5	0.0033	0.0015	0.0014	0.0015	0.0014	0.0025	0.0014	0.0020	0.0016	0.0018
q=−0.4	0.0029	0.0013	0.0012	0.0012	0.0013	0.0019	0.0014	0.0016	0.0014	0.0014
q=−0.3	0.0017	0.0011	0.0011	0.0011	0.0011	0.0016	0.0012	0.0014	0.0012	0.0012
q=−0.2	0.0014	0.0010	0.0011	0.0011	0.0010	0.0012	0.0010	0.0012	0.0011	0.0010
q=−0.1	0.0010	0.0009	0.0010	0.0010	0.0010	0.0011	0.0009	0.0010	0.0010	0.0009
q=0	0.0009	0.0009	0.0009	0.0009	0.0009	0.0009	0.0009	0.0009	0.0009	0.0009
q=0.1	0.0006	0.0008	0.0008	0.0008	0.0008	0.0009	0.0008	0.0006	0.0009	0.0008
q=0.2	0.0005	0.0007	0.0007	0.0008	0.0007	0.0007	0.0008	0.0005	0.0008	0.0006
q=0.3	0.0004	0.0008	0.0007	0.0006	0.0006	0.0004	0.0008	0.0003	0.0008	0.0006
q=0.4	0.0003	0.0008	0.0007	0.0005	0.0005	0.0003	0.0008	0.0003	0.0008	0.0005
q=0.5	0.0002	0.0008	0.0007	0.0005	0.0004	0.0003	0.0009	0.0003	0.0007	0.0005
q=0.6	0.0002	0.0009	0.0006	0.0003	0.0004	0.0002	0.0014	0.0003	0.0006	0.0003
q=0.7	0.0003	0.0008	0.0005	0.0003	0.0004	0.0001	0.0016	0.0002	0.0005	0.0003
q=0.8	0.0003	0.0008	0.0004	0.0002	0.0004	0.0000	0.0019	0.0003	0.0004	0.0003
q=0.9	0.0004	0.0010	0.0003	0.0001	0.0004	0.0000	0.0015	0.0002	0.0003	0.0003
q=1.1	0.0003	0.0021	0.0002	0.000	0.0003	0.0000	0.0015	0.0002	0.0002	0.0002
q=1.2	0.0003	0.0028	0.0002	0.0000	0.0002	0.0000	0.0015	0.0002	0.0001	0.0002
q=1.3	0.0002	0.0030	0.0002	0.0000	0.0002	0.0000	0.0015	0.0001	0.0000	0.0001
q=1.4	0.0002	0.0028	0.0001	0.0000	0.0003	0.0000	0.0014	0.0001	0.0000	0.0001
q=1.5	0.0003	0.0029	0.0001	0.0000	0.0004	0.0000	0.0011	0.0000	0.0000	0.0000
q=1.6	0.0003	0.0025	0.0001	0.0000	0.0006	0.0000	0.0010	0.0000	0.0000	0.0000
q=1.7	0.0003	0.0022	0.0001	0.0000	0.0008	0.0000	0.0008	0.0000	0.0000	0.0000
q=1.8	0.0004	0.0022	0.0001	0.0000	0.0010	0.0000	0.0008	0.0000	0.0000	0.0000
q=1.9	0.0004	0.0018	0.0000	0.0000	0.0018	0.0000	0.0006	0.0000	0.0000	0.0000
q=2	0.0004	0.0018	0.0001	0.0000	0.0023	0.0000	0.0006	0.0000	0.0000	0.0000

**Table 3:** Mapping of EEG channels to ordinal labels (C1–C19).

Channel	Label	Channel	Label
Fp1-Av	C1	C4-Av	C11
Fp2-Av	C2	T4-Av	C12
F7-Av	C3	T5-Av	C13
F3-Av	C4	P3-Av	C14
Fz-Av	C5	Pz-Av	C15
F4-Av	C6	P4-Av	C16
F8-Av	C7	T6-Av	C17
T3-Av	C8	O1-Av	C18
C3-Av	C9	O2-Av	C19
Cz-Av	C10		

**Table 4:** Random forest model results. The results were consistent in all of the four different setups. Validation was performed using 10-fold cross validation.

Class	TP Rate	FP Rate	Precision	Recall	F-Measure	MCC	ROC Area	PRC Area
TD	1.000	0.041	0.951	1.000	0.975	0.955	1.000	1.000
ASD	0.959	0.000	1.000	0.959	0.979	0.955	1.000	1.000
Weighted Avg.	0.977	0.018	0.978	0.977	0.977	0.955	1.000	1.000

**Table 5:** J48 decision tree algorithm results with 57 attributes and top five attributes based on gain-ratio. Validation was performewd using 10-fold cross validation.

Class	TP Rate	FP Rate	Precision	Recall	F-Measure	MCC	ROC Area	PRC Area
TD	0.974	0.041	0.950	0.974	0.962	0.931	0.963	0.927
ASD	0.959	0.026	0.979	0.959	0.969	0.931	0.963	0.962
Weighted Avg.	0.966	0.032	0.966	0.966	0.966	0.931	0.963	0.946

**Table 6:** SVM model results from the setup with all 57 attributes. Validation was performed using 10-fold cross validation.

Class	TP Rate	FP Rate	Precision	Recall	F-Measure	MCC	ROC Area	PRC Area
TD	0.974	0.041	0.950	0.974	0.962	0.931	0.993	0.991
ASD	0.959	0.026	0.979	0.959	0.969	0.931	0.993	0.994
Weighted Avg.	0.966	0.032	0.966	0.966	0.966	0.931	0.993	0.993

**Table 7:** RepTree decision tree algorithm results from the setup with the top five attributes. Validation was conducted using 10-fold cross validation.

Class	TP Rate	FP Rate	Precision	Recall	F-Measure	MCC	ROC Area	PRC Area
TD	0.974	0.041	0.950	0.974	0.962	0.931	0.948	0.913
ASD	0.959	0.026	0.979	0.959	0.969	0.931	0.948	0.943
Weighted Avg.	0.966	0.032	0.966	0.966	0.966	0.931	0.948	0.930

**Table 8:** ANN model results from the setup with all 57 attributes. Validation was performed using 10-fold cross validation.

Class	TP Rate	FP Rate	Precision	Recall	F-Measure	MCC	ROC Area	PRC Area
TD	0.949	0.061	0.925	0.949	0.937	0.885	0.978	0.980
ASD	0.939	0.051	0.958	0.939	0.948	0.885	0.978	0.979
Weighted Avg.	0.943	0.056	0.944	0.943	0.943	0.885	0.978	0.979

## Data Availability

The data presented in this study are available on request from the corresponding author due to legal reasons.
